# Inhibition of cytokinesis by wiskostatin does not rely on N-WASP/Arp2/3 complex pathway

**DOI:** 10.1186/1471-2121-9-42

**Published:** 2008-07-30

**Authors:** Guillaume Bompard, Gabriel Rabeharivelo, Nathalie Morin

**Affiliations:** 1 CRBM, CNRS UMR 5237, Université Montpellier I et II, 1919 route de Mende, 34293 Montpellier Cedex 5, France

## Abstract

**Background:**

Cytokinesis is the final step of cell division taking place at the end of mitosis during which the cytoplasmic content and replicated chromosomes of a cell are equally partitioned between the two daughter cells. This process is achieved by the formation and the ingression of an actomyosin contractile ring under the control of equatorial microtubules. The mechanisms of contractile ring formation are not fully understood but involve recruitment of preexisting actin filaments and *de novo *actin polymerisation.

**Results:**

In this study, we evaluated the role of the actin nucleation factor, Arp2/3 complex, during cytokinesis. We found that the Arp2/3 complex is recruited late to the cleavage furrow suggesting a potential involvement of Arp2/3 complex during this process. Furthermore, wiskostatin a potent inhibitor of N-WASP activity towards the Arp2/3 complex blocked cytokinesis without affecting mitosis. Nonetheless, this inhibition could not be reproduced using alternative approaches targeting the N-WASP/Arp2/3 complex pathway.

**Conclusion:**

We conclude that the wiskostatin induced defective cytokinesis does not occur through the inhibition of the N-WASP/Arp2/3 pathway. Wiskostatin is likely to either directly target other proteins required for cytokinesis progression or alternately wiskostatin bound to N-WASP could affect the activity of other factors involved in cytokinesis.

## Background

Cytokinesis is essential to cell growth and has to be tightly regulated to insure equal distribution of the cytoplasm content and newly replicated chromosomes within two daughter cells at the end of mitosis. Defective chromosome segregation or daughter cell separation can alter chromosome number, which is commonly found in tumour cells and potentially involved in cancer development [1]. For these reasons it is essential to understand the signalling pathways that coordinate mitotic exit and cytokinesis.

In metazoans, the central spindle forms during anaphase between the two sets of chromosomes. Simultaneously, the cleavage furrow composed by actin and myosin II filaments is established at the cell equator under the control of peripheral microtubules (central spindle and/or peripheral astral microtubules) [2]. Thus, constriction of the actomyosin ring can only start once the two sets of chromosomes are segregated, insuring genome integrity. The two daughter cells stay connected by a cytoplasmic bridge with the *midbody *in the centre for several hours until the bridge is finally severed during abscission [3].

The small GTPase RhoA has been shown to play a major role in cleavage furrow establishment. Indeed, the GTPase exchange factor (GEF) ECT2 is specifically recruited at the cell equator under the control of the *centralspindlin *complex (MKLP1/MgcRacGAP) also required for central spindle formation [3-7]. Once activated, RhoA induces, through its effector ROCK, the phosphorylation of myosin regulatory light chain (MRLC), which is necessary for contractile ring formation and ingression [3].

In mammals, actin filaments present in the contractile ring derive from preexisting F-actin flux and *de novo *actin polymerisation [8]. In *S. pombe*, cleavage furrow establishment and activity depend on *de novo *actin polymerisation driven by the two main actin nucleation factors: the Arp2/3 complex and a formin (Cdc12) [9,10]. Arp2/3 complex is activated by Myo1 and WSP1 at the equator and is involved in the maturation and/or the maintenance of the cleavage furrow [11,12]. In mammals the mDia formins, orthologs of Cdc12 and RhoA effectors, are required for cytokinesis [13,14]. In agreement with a conserved mechanism for cleavage furrow formation and activity, the Arp2/3 complex and its activator WASP/N-WASP, ortholog of WSP1, were found to potentially play a role during cytokinesis [15-19].

In this study the functional role of the Arp2/3 complex during cytokinesis in mammalian cells was evaluated. We demonstrated that Arp2/3 complex is recruited late to the cleavage furrow. Consequences of the inhibition of WASP/N-WASP activity towards the Arp2/3 complex during cytokinesis were then studied using the newly identified inhibitor: wiskostatin [20]. We show that wiskostatin strongly inhibits cytokinesis inducing cell binucleation without affecting the cell cycle. However, these results are unlikely related to the Arp2/3 complex function since the wiskostatin induced phenotype could not be validated using N-WASP and/or Arp2/3 RNA interference approach. This novel effect of wiskostatin on cytokinesis is also not related to another known unexpected effect of this compound i.e. ATP depletion [21].

This study that was originally designed to study N-WASP/Arp2/3 complex pathway during cytokinesis, unexpectedly highlighted a novel effect of wiskostatin. For this reason, wiskostatin will have to be used with caution in future experiments.

## Results

### Localisation of Arp2/3 complex during mitosis

**Figure 1:**
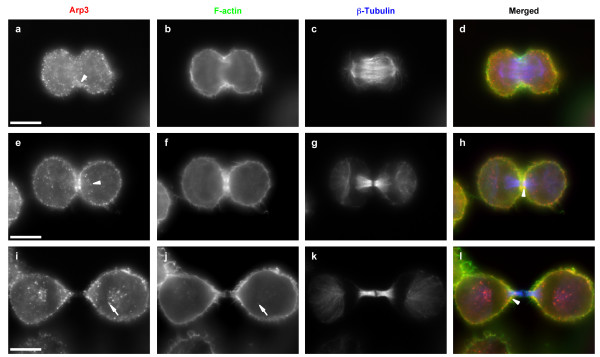
**Arp3 localisation during mitosis and cytokinesis**. HeLa cells were enriched in mitosis after thymidine and RO3306 blocks (see Methods for details). Two hours after release from RO3306 block cells were permeabilised and fixed. Cells were treated for indirect Alexa 555 localisation of Arp3 (a, e, and i) and Alexa 350 localisation of β-tubulin (c, g and k) with specific antibodies. F-actin was visualised with FITC-coupled phalloidin (b, f, and j). Merged images are presented (d, h and l). Images are representative of the different stages of mitosis: anaphase (a-d), late telophase (e-h) and cytokinesis (i-l). Bars, 10 μm.

In mammalian cells, the localisation of endogenous Arp2/3 complex during mitosis is currently unknown. In order to study the function of the N-WASP/Arp2/3 complex pathway during cytokinesis, we thus analyzed the subcellular localisation of Arp3 and p34 (ARPC2) subunits of the Arp2/3 complex by indirect immunofluorescence. In interphase HeLa cells using monoclonal anti-Arp3 antibody, the Arp2/3 complex was found to be diffuse and associated with vesicle-like structures in the cytoplasm but also accumulated in cortical F-actin-rich structures, which are likely lamellipodia (data not shown). To better visualize cytoskeleton bound Arp2/3 complex in mitotic cells, the cytosolic unbound fraction was eliminated by permeabilising the cells prior to fixation (Fig. 1). Under these conditions, Arp3 presented a diffuse vesicular-like localisation and was not particularly enriched into the nascent cleavage furrow during anaphase (Fig. 1, a, arrowhead). However, during late telophase, once the cleavage furrow is fully contracted, Arp3 was strongly enriched in the close vicinity of the contractile ring, as visualized by the F-actin staining (Fig. 1, h, arrowhead). Furthermore, the vesicular-like localisation of Arp3 was organised around the minus-end and along microtubules (Fig. 1, e, arrowhead). In cytokinesis, when the cytoplasmic bridge between the two daughter cells extended, Arp3 colocalised with F-actin in the cortex facing the cytoplasmic bridge (Fig. 1, l, arrowhead) and within vesicle-like structures (Fig. 1, i and 1j, arrows). Furthermore, the Arp2/3 complex also localised within the cytoplasmic bridge but not in the *midbody *(see Additional file 1). This subcellular localisation of Arp2/3 complex was further confirmed by using a polyclonal antibody directed against p34 subunit (see Additional file 1). Thus, the localization of Arp2/3 complex is in agreement with a potential functional role during cytokinesis.

### Wiskostatin inhibits cytokinesis but not mitosis

The mammalian ortholog of WSP1, WASP/N-WASP, is regulated by autoinhibition essentially relieved by binding of activated small GTPase Cdc42 and/or phosphatidyl inositol 4,5-biphosphate (PtdIns[4,5]P_2_) [10,22]. GTP loaded Cdc42 binds to the GBD (GTPase binding domain) of WASP/N-WASP, which is preceded by a basic region where PtdIns[4,5]P_2 _interacts [22]. Wiskostatin is a recently identified WASP/N-WASP chemical inhibitor designed to bind to the GBD [20]. Wiskostatin not only prevents Cdc42 binding, but also stabilises the autoinhibited state of this actin nucleation promoting factor [20]. This led us to use wiskostatin to investigate the role of the Arp2/3 complex activated by WASP/N-WASP during cytokinesis.

**Figure 2:**
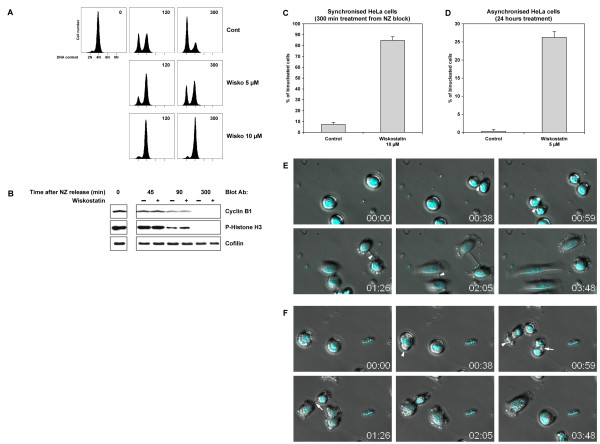
**Wiskostatin inhibits cytokinesis but not mitosis**. A. HeLa cells were synchronised in prometaphase with nocodazole and treated with vehicle or indicated concentrations of wiskostatin. Cells were harvested at indicated time points and DNA content determined by FACS analysis. A similar scale was used for each histogram. This result is representative of at least 3 independent experiments. B. HeLa cells synchronised as previously described were treated with vehicle (-) or 10 μM wiskostatin (+) and harvested at indicated time points. Cyclin B1, phosphorylation of Ser 10 of histone H3 and cofilin levels were determined by immunoblot analysis with specific antibodies. This result is representative of at least 5 independent experiments. C. HeLa cells stably expressing GFP-tagged histone H2B were synchronised as previously described, treated with vehicle (Control) or 10 μM wiskostatin (Wiskostatin 10 μM) and fixed after 300 minutes. F-actin was stained and nuclei per cell determined. Percentage of binucleated cells was then calculated. 536 cells treated with vehicle and 569 cells treated with wiskostatin were count from three independent experiments. Error bars represent standard error of the mean (SEM). D. Assynchronous HeLa cells stably expressing GFP-tagged histone H2B were treated with vehicle (Control) or 5 μM wiskostastin (Wiskostatin 5 μM) for 24 hours and were then fixed. Percentages of binucleated cells were determined as previously indicated. 629 cells treated with vehicle and 640 cells treated with wiskostatin were count from three independent experiments. Error bars represent SEM. Representative images from time lapse movies of HeLa cells stably expressing GFP-tagged histone H2B synchronised as previously described, treated with vehicle (E) or 10 μM wiskostatin (F). Images represent merged between DIC and fluorescent (Histone H2B) images. Time indicated as hours: minutes.

HeLa cells stably expressing GFP-tagged histone H2B (HeLa GFP-H2B) were synchronised in prometaphase with nocodazole and were released for various times after shake-off in presence of vehicle (DMSO) or increasing concentration of wiskostatin (from 1 – 10 μM). Cell DNA content was determined by flow cytometry at 0, 120 and 300 minutes after nocodazole release. In control conditions, at T = 0, cells had a 4N content confirming mitosis synchronisation mediated by nocodazole. 120 minutes later half of the cells had exit mitosis and displayed a 2N content. Finally, 300 min after release, control cells were mostly in G1 phase (diploid cells, 2N) (Fig. 2A). In contrast, under wiskostatin treatment, the number of tetraploid cells (4N) was abnormally elevated at 120 min after release suggesting a mitotic delay both at 5 and 10 μM doses (Fig. 2A). 300 minutes after nocodazole release under 5 μM wiskostatin, half of the cells had a 2N content while under 10 μM wiskostatin treatment all cells were tetraploid indicating that they did not proceed through mitosis (Fig. 2A). Thus wiskostatin inhibits mitotic progression in a dose dependent manner.

The increased number of tetraploid cells under wiskostatin could either result from an inhibition of mitosis or cytokinesis. To answer this question, we first analysed the status of cyclin B1, which is degraded at the metaphase/anaphase transition. Mitotic HeLa cells were released in presence of vehicle alone or wiskostatin (10 μM), and cyclin B1 levels were determined by immunoblot analysis (Fig. 2B). No difference in the kinetics of cyclin B1 degradation was observed between control and wiskostatin treated cells. This result was further confirmed by studying another mitotic marker, the phosphorylation of histone H3 on serine 10 (Ser 10) which correlates with chromatin condensation [23]. As expected, histone H3 phosphorylation was not affected by wiskostatin treatment (Fig. 2B). Equal sample loading was confirmed by cofilin immunoblot analysis (Fig. 2B). These results clearly demonstrate that wiskostatin does not inhibit mitotic progression.

Taken altogether, our results indicate an effect of wiskostatin on cytokinesis. Cytokinesis failure is generally associated with cell binucleation. To investigate this hypothesis, nocodazole synchronised HeLa GFP H2B cells were grown in presence of vehicle alone or wiskostatin (10 μM), fixed after 300 minutes and binucleated cells were quantified. In control condition, around 7% of cells were found to be binucleated (7.26% SEM ± 2.11, n = 536). In contrast, 85% of wiskostatin treated cells contained two nuclei (84.79% SEM ± 3.44, n = 569) (Fig. 2C). To rule out any cooperative effects of nocodazole and wiskostatin, asynchronous HeLa GFP-H2B cells were grown in presence of 5 μM wiskostatin for 24 hours and binucleated cells were counted as previously described. Under these conditions more than 26% of cells were found to be binucleated (26.24%, SEM ± 0.92, n = 640) compared to 0.3% (0.3%, SEM ± 0.3, n = 629) of cells treated with vehicle alone (Fig. 2D). Similar results were obtained with NIH3T3 and XL2 cells (data not shown). This result clearly demonstrates that wiskostatin affects mitotic exit.

In order to analyse wiskostatin induced cytokinesis defects in more details, time-lapse microscopy was used. In vehicle treated HeLa GFP-H2B cells cytokinesis started around 45 minutes after release from nocodazole block (Fig. 2E, see Additional file 2). After complete cleavage furrow contraction, daughter cells were linked to each other by a cytoplasmic bridge, which extended over time with the *midbody *in its centre (Fig. 2E, arrowheads). Finally, after several hours, abscission occurred. Under wiskostatin treatment, cytokinesis started with the same kinetics as control cells (Fig. 2F, see Additional file 3) but prior and during cleavage furrow contraction blebs were visible at cell poles (Fig. 2F, arrowheads). Finally, once the cleavage furrow fully contracted, the cytoplasmic bridge did not extend, the contractile ring relaxed and daughter cells finally fused-back together (Fig. 2F, arrows).

**Figure 3:**
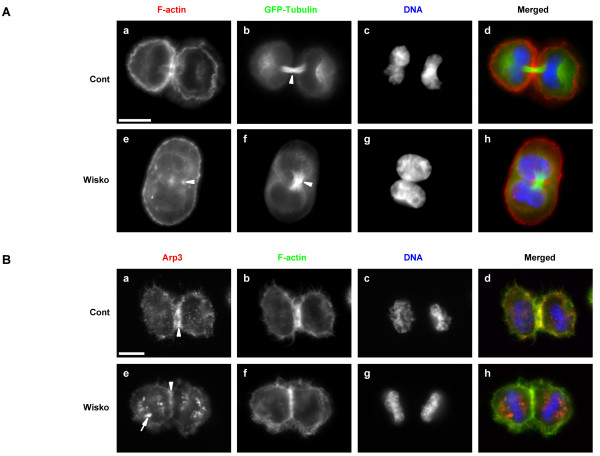
**Wiskostatin inhibits late cytokinesis**. A. Wiskostatin does not affect microtubule organisation. HeLa cells stably expressing GFP-tagged β-tubulin were synchronised in prometaphase with nocodazole and released after a shake-off in presence of vehicle (Cont) or 10 μM wiskostatin (Wisko). 120 minutes after release cells were fixed and F-actin (a and e) and DNA (c and g) stained respectively with Alexa 555-coupled phalloidin and DAPI. β-tubulin (b and f) was directly visualised. Merged images (d and h) are presented. B. Wiskostatin alters Arp3 localisation. HeLa cells were synchronised in G2/M by thymidine and RO3306 double block. 120 minutes after release cells were fixed and stained as described in Figure 1. Images a and e represent staining of Arp3, b and f staining of F-actin and c and g staining of β-tubulin. Merged images (d and h) are presented. Bars, 10 μm.

We then studied the organisation of the cytoskeleton in more details. Mitotic HeLa cells stably expressing GFP-tagged β-Tubulin were fixed two hours after nocodazole release in presence of vehicle or 10 μM wiskostatin, and were stained for F-actin and DNA. During cytokinesis, the microtubule bundles of the central spindle, which are not associated with kinetochores, are compacted by the contraction of the cleavage furrow. In control cells, these compacted bundles were clearly visible between the two daughter cells (Fig. 3A, b, arrowhead). Similarly in wiskostatin treated cells, compacted microtubule bundles were also present between newly assembled nuclei but cells were binucleated (Fig. 3A, f, arrowhead). Cleavage furrow relaxation induced by wiskostatin was not associated with obvious F-actin reorganisation and some cleavage furrow remnant could be observed surrounding the central spindle as evidenced by F-actin staining (Fig. 3A, e, arrowhead). Relocalisation of ECT2 from the central spindle in anaphase to the *midbody *during cytokinesis was also not altered by wiskostatin treatment (data not shown) confirming that wiskostatin did not affect cleavage furrow formation and initial contraction.

We next studied the localisation of Arp3 during cytokinesis under wiskostatin treatment. As previously showed, Arp3 was enriched within the cleavage furrow once fully contracted in vehicle treated cells at the end of telophase (Fig. 3B, a, arrowhead). Under wiskostatin treatment, Arp3 localisation within the contractile ring was reduced (Fig. 3B, e, arrowhead). In addition, wiskostatin induced the clustering of Arp3 within vesicle-like structures throughout the cell (Fig. 3B, e, arrow).

Altogether, our results showed that wiskostatin inhibited late cell cycle events required for abscission to occur. Wiskostatin induced cleavage furrow regression was associated with a reduced localisation of Arp2/3 complex within the contractile ring.

### Wiskostatin effects onto cytokinesis are not reproduced by alternative inhibition of the N-WASP/Arp2/3 pathway

**Figure 4:**
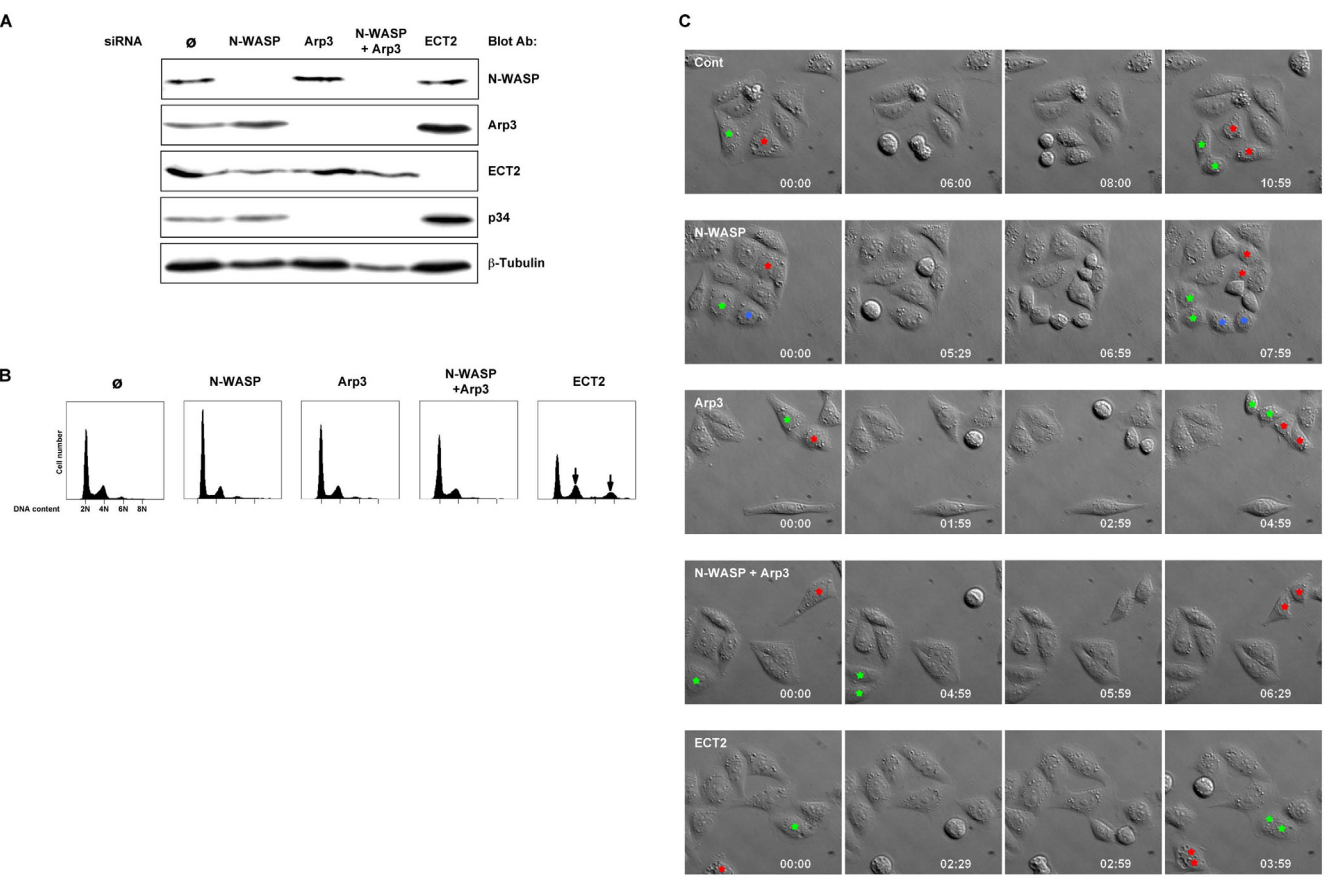
**Arp3 and/or N-WASP knockdowns do not affect cytokinesis**. A. HeLa cells were transfected with water (Ø) or specific siRNA directed against N-WASP or Arp3 or N-WASP + Arp3 or ECT2. 72 hours after transfection cells were harvested and lysed. N-WASP, Arp3, ECT2, p34 and β-tubulin protein levels were determined by immunoblot analysis using specific antibodies. B. The DNA content of the same HeLa cells was determined by flow cytometry analysis. Arrows indicate abnormal DNA content in ECT2 knockdown cells. A similar scale was used for each histogram. These results are representative of at least three independent experiments. C. Representative DIC images from time-lapse movies of HeLa cells transfected with water (Ø), siRNA directed against N-WASP (N-WASP), against Arp3 (Arp3), against Arp3 and N-WASP (Arp3 + N-WASP) or against ECT2. Images started to be acquired 24 hours post-transfection for 40 hours. Coloured stars indicate the nuclei of dividing cells and of their daughter cells. Time indicated as hours: minutes.

In order to study whether the mislocalisation of Arp3, induced by wiskostatin, was responsible for defective cytokinesis, we used alternative approaches to inhibit N-WASP and Arp2/3 complex activities. We first knocked-down N-WASP and Arp3 protein levels using an siRNA approach. N-WASP and Arp3 expressions were strongly reduced in cells treated for 72 hours with specific siRNA (more than 80% inhibition) as demonstrated by immunoblot analysis (Fig. 4A). As previously shown, Arp3 knockdown is associated with down-regulation of other Arp2/3 subunits [24] as revealed by the strong inhibition of p34 expression in Arp3 null cells (Fig. 4A). The DNA content of siRNA treated cells was then evaluated by flow cytometry in order to study the consequences of N-WASP and/or Arp3 depletion on cytokinesis. No major differences between control cells and cells transfected with N-WASP and/or Arp3 siRNAs were observed (Fig. 4B). In contrast, tetraploid and also octaploid cells accumulated when transfected with siRNA inhibiting ECT2 protein expression (Fig. 4A, more than 90% inhibition), a major effector of cytokinesis (Fig. 4B, arrows). The phenotypes resulting from siRNA transfection were also evaluated by indirect immunofluorescence analysis. Cellular morphology, F-actin organisation and Arp3 staining were not affected in control and N-WASP knockdown cells (see Additional file 4). In contrast, Arp3 staining was greatly reduced in cells transfected with Arp3 siRNA for 72 hours. The morphology of these cells was altered as they appeared rounder and smaller, a phenotype likely due to the disorganization of the F-actin network (see Additional file 4). During cytokinesis, Arp3 recruitment to the contractile ring was only reduced in Arp3 null cells but not in control and N-WASP null cells (see Additional file 4). We obtained similar results using other siRNAs targeting N-WASP and Arp3 sequences (data not shown). Our data suggest that Arp2/3 complex localisation within the cleavage furrow does not rely on N-WASP and is not mandatory for cytokinesis to occur.

In order to investigate possible subtle consequences of N-WASP and/or Arp3 losses on mitosis/cytokinesis, siRNA treated cells were studied by time-lapse microscopy. The time-lapse experiment began 24 hours after siRNA transfection when protein expression was significantly reduced (see Additional file 4) and cells were imaged every 30 minutes for 30 hours. No differences in terms of frequency and kinetics of mitosis and cytokinesis were observed between control cells (Fig. 4C and see Additional file 5), N-WASP null-cells (Fig. 4C and see Additional file 6), Arp3 null-cells (Fig. 4C and see Additional file 7), N-WASP and Arp3 null-cells (Fig. 4C and see Additional file 8). In contrast, ECT2 knockdown strongly interfered with cell division inducing binucleation and tetranucleation over time (Fig. 4C and see Additional file 9). Surprisingly, these results suggest that the loss of N-WASP and/or Arp2/3 complex, are not associated with cytokinesis failure as we were expecting based on the effects of wiskostatin. In order to confirm these unexpected results, various N-WASP mutants known to interfere with Arp2/3 complex activity were overexpressed and their effects on cytokinesis evaluated. WASP family proteins are characterized by the presence, at the carboxy-terminus, of three independent domains: the verprolin homology (V) (a.k.a. WASP homology 2/WH2 domain), central (C) and acidic (A) regions. These domains form the so-called VCA module, which is necessary and sufficient to activate Arp2/3-dependent actin polymerisation *in vitro*. The V and A regions bind actin monomers and Arp2/3 respectively, whilst the C region binds Arp2/3 and induces crucial changes in the tertiary and quaternary structures of the Arp2/3 complex, thereby regulating the ability of the Arp2/3 complex to induce actin polymerisation [10]. Deletion of the A region within the sequence of WASP family proteins compromises binding to the Arp2/3 complex. This mutation acts as a dominant negative protein. On the other hand, overexpression of VCA and CA modules from WASP family proteins strongly induces mislocalised Arp2/3 complex activation and unproductive Arp2/3 binding respectively [25,26]. These constructs titrate endogenous Arp2/3 complex and inhibit F-actin processes depending onto Arp2/3 activity [10]. Vector expressing GFP, GFP-tagged N-WASP, N-WASP ΔA (deletion of the acidic domain), VCA or CA modules from N-WASP were transfected in NIH3T3 fibroblasts. Three days after transfection cells were fixed and binucleated transfected cells counted. Results from a representative experiment are presented in Table 1. As shown, no major differences were observed between GFP, GFP-tagged N-WASP, N-WASP ΔA and CA overexpressing cells, although, overexpression of GFP-tagged VCA slightly, but consistently, increased cell binucleation (Table 1).

**Table 1:** Effect of various N-WASP constructs onto cell ploidy.

Vector	Total cell number	Number of binucleated cells	% of binucleated cells
GFP	419	17	4.06
GFP VCA	527	61	11.57
GFP CA	629	30	4.77
GFP N-WASP	351	19	5.41
GFP N-WASP ΔA	130	7	3.38

NIH3T3 cells were transfected with indicated constructs. Three days after transfection cells were harvested and a fifth seeded onto glass coverslips. Two hours after seeding cells were fixed. Nuclei number of transfected cells was determined after F-actin and DNA staining respectively with TRITC-coupled phalloidin and DAPI. Total cell number and the number of binucleated cells from a representative experiment are presented as well as the deduced percentage of binucleated cells.

Altogether, our data demonstrated that the inhibition of N-WASP/Arp2/3 pathway is not sufficient to mimic wiskostatin effects on cytokinesis. Therefore, it is likely that wiskostatin affect the activity of additional targets involved in cytokinesis.

### Wiskostatin effect onto cytokinesis is independent of ATP depletion

**Figure 5:**
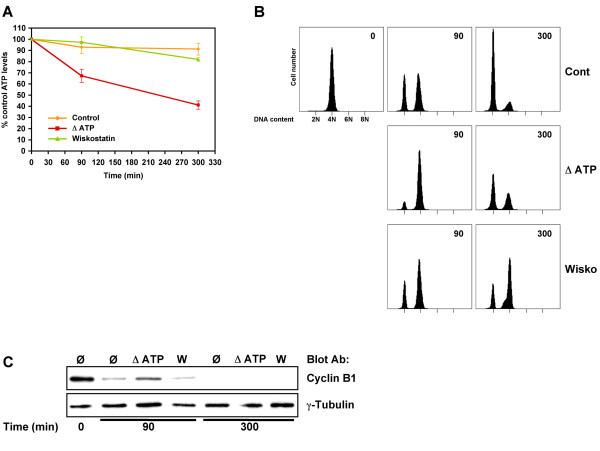
**Wiskostatin does not affect cellular ATP levels**. A. Cellular ATP levels in wiskostatin treated cells. HeLa cells were synchronised in prometaphase with nocodazole and released after shake-off in presence of vehicle (control), 10 mM NaN_3 _+ 6 mM deoxyglucose (ΔATP) or 10 μM wiskostatin (wiskostatin). After indicated time points cells were harvested and lysed in boiling water for 10 minutes. Cellular ATP contents were determined on clarified lysates using ATP determination kit (see Methods). Cellular ATP level at time point 0 from each treatment was defined as 100%. Graph represents mean of three independent experiments. Error bars represent SEM. ATP depletion delays mitosis. B. HeLa cells synchronised and treated as previously described were harvested at indicated time points and DNA content analysed by flow cytometry. C. In similarly treated cells cyclin B1 and γ-tubulin levels were determined by immunoblot analysis using specific antibodies.

Taken altogether, our results suggest that wiskostatin effects on cytokinesis progression cannot solely be explained by the inhibition of N-WASP. During the course of our study, wiskostatin was shown to induce cellular ATP depletion [21]. We thus evaluated whether ATP depletion may interfere with cytokinesis progression. Mitotic HeLa cells released in presence of vehicle or wiskostatin (10 μM) from a nocodazole block were harvested at various time points and lysed for 10 minutes at 100°C in distilled water. ATP levels were then determined from clarified lysates using a bioluminescent luciferase assay. In vehicle (at 300 min, 91.2%, SEM ± 5.3, n = 3) or wiskostatin (at 300 min, 82%, SEM ± 1.5, n = 3) treated cells no major variation of ATP levels was observed during mitosis/cytokinesis (Fig. 5A). In contrast, ATP levels strongly decreased in cells released in presence of energy poisons such as sodium azide and 2-deoxy-D-glucose (at 300 min, 41.1%, SEM ± 3.8, n = 3, Fig. 5A).

In order to confirm that inhibition of cytokinesis induced by wiskostatin was independent of ATP depletion, DNA content of HeLa cells synchronised and treated as mentioned was determined by flow cytometry. Whereas vehicle or ATP depleted treated cells were mainly diploids after 300 minutes from nocodazole release, wiskostatin treated cells were massively tetraploids and likely binucleated (Fig. 5B). Interestingly, we observed a delay in exit to mitosis in ATP depleted cells (Fig. 5B, see 90 min time point), which was correlated with a delay of cyclin B1 degradation as demonstrated by immunoblot analysis (Fig. 5C). As previously demonstrated, there was no effect of wiskostatin on cyclin B1 degradation. Time-lapse microscopy confirmed that ATP depletion delayed mitotic exit whereas wiskostatin did not (data not shown).

Our results showed that ATP depletion does not block mitosis/cytokinesis but simply delays it. Furthermore, we established that inhibition of cytokinesis by wiskostatin is independent of ATP depletion suggesting additional targets for wiskostatin.

## Discussion

Partitioning of the two daughter cells following cytokinesis is crucial to allow faithful segregation of chromosomes. Disrupting the final stages of cytokinesis can lead to multinucleated cells and genome instability. The involvement of the actin cytoskeleton during cytokinesis is now well established. The cleavage furrow is formed during anaphase by actin and myosin filaments and its complete ingression allows daughter cell separation at the end of mitosis. However, little data regarding actin dynamics during cytokinesis is available. A recent study showed that actin filaments present within the cleavage furrow of mammalian cells come from the flux of preexisting F-actin and *de novo in situ *actin polymerisation [8]. However the functional importance of *de novo *actin polymerisation or the identity of the nucleation factor involved remain unanswered questions.

In *S. pombe*, the two main actin nucleation factors, the Arp2/3 complex and a formin, are required for cleavage furrow establishment and activity [9,11]. In mammals, the involvement of formins during cytokinesis is now well established suggesting a conserved mechanism between yeast and mammals for cleavage furrow formation [3,13,14,27]. However, in metazoan the involvement of WASP/N-WASP and the Arp2/3 complex during cytokinesis is still a matter of debate. Very little information regarding the function of WASP/N-WASP during cell growth in general and cytokinesis in particular are available. In nematodes, knockdown of WSP-1 protein, the WASP/N-WASP ortholog, leads to cytokinesis defects [18]. N-WASP knockout in mouse is embryonic lethal (E12) and primary fibroblasts isolated from knockout embryos are unable to grow without being transformed suggesting an involvement of N-WASP in cell growth [28,29]. Data regarding the involvement of the Arp2/3 complex during cytokinesis in metazoans are more controversial. In nematode, the requirement of the Arp2/3 complex is unclear since two studies using RNA interference lead to conflicting results. Two groups described that knocking-down most of the Arp2/3 complex subunits lead to embryonic lethality, however only the group of Skop et al. showed that these depletions were associated with early or late cytokinesis defects [16,30]. In Drosophila, the picture is more readable since Arp3 knockdown in S2 cells led to cell binucleation [15]. Furthermore, the Arp2/3 complex is required for the assembly of two Drosophila embryonic structures highly related to the cleavage furrow, the pseudocleavage furrow in syncytial embryos and the ring canal [31,32]. Finally, in mammals, p21 (ARPC3) subunit of the Arp2/3 complex is an essential gene, as knockdown results in a block in cell growth phenotype [33]. However, a potential relation with a cytokinesis defect was not reported.

In summary, the data available in the literature on the functional importance of the Arp2/3 complex for cell growth are clear, however because of the pleïotropic functions of the actin cytoskeleton, specific requirements of Arp2/3 complex activity for efficient cytokinesis are still questionable.

In this study, we showed that endogenous Arp3, despite being localised to the cell cortex, did not accumulate in the nascent contractile ring. However, once the cleavage furrow was completely contracted during cytokinesis, Arp2/3 complex colocalised with F-actin within this structure. Arp2/3 complex was also found within the cytoplasmic bridge linking daughter cells at the end of mitosis in agreement with the biochemical association of Arp2/3 subunits with the central spindle [16]. The absence of early specific localisation of Arp3 within the cleavage furrow suggests that the Arp2/3 complex is not involved in its establishment. This is in agreement with the proposed role of the Arp2/3 complex in fission yeast where it is recruited late to/around the contractile ring and seems to be involved in cleavage furrow maintenance by allowing new plasma membrane addition [12]. New membrane addition relies on vesicle recruitment and is essential for cleavage furrow ingression and to seal up daughter cells at time of abscission [34]. The Arp2/3 complex is involved in endocytosis, vesicular trafficking but also in the polarised delivery of certain proteins to the plasma membrane [35,36]. All these processes are known or supposed to be important during cytokinesis [34]. We showed that Arp2/3 complex was enriched in vesicle-like structure localised on microtubules facing the cytoplasmic bridge between daughter cells.

We found that inhibition of N-WASP activity towards the Arp2/3 complex using wiskostatin strongly impaired cytokinesis without affecting mitosis in a dose dependent manner. Wiskostatin inhibited final stages of cytokinesis, which correlated with a decrease of the Arp2/3 complex from the contractile ring and the clustering of the Arp2/3 complex within vesicle-like structures. The late inhibition of cytokinesis by wiskostatin suggests that the Arp2/3 complex is required at the end of cytokinesis and/or for abscission in agreement with its late recruitment within the cleavage furrow. However, we were unable to confirm these results by knocking-down N-WASP and/or Arp3 protein expression by RNA interference. Moreover, N-WASP depletion showed that Arp3 localisation within the cleavage furrow is not dependent upon N-WASP. Different possibilities can explain the discrepancy between the results we obtained using RNA interference versus wiskostatin. First, we cannot exclude that remaining N-WASP and/or Arp3 proteins, in siRNA treated cells, are sufficient to trigger cytokinesis bearing in mind that complete depletion of these proteins is associated with cell growth defects and embryonic lethality [29-31,33]. Second, wiskostatin could have additional targets related or not to N-WASP. Wiskostatin binds purified N-WASP *in vitro *but nothing is known about binding to native N-WASP. Native N-WASP is predominantly associated with WIP (WASP-interacting protein), a protein involved in N-WASP inhibition but also required for N-WASP activation [37-39]. WIP can regulate F-actin organisation independently of its effects on N-WASP [39]. Wiskostatin could prevent the release or the binding of WIP or other unknown proteins. This could have major consequences for the progression of cytokinesis if such proteins play a role during this process. Furthermore, this could explain the discrepancy between wiskostatin and N-WASP knockdown results. Finally, wiskostatin could inhibit additional targets unrelated to N-WASP. Indeed, wiskostatin was recently shown to deplete cellular ATP in an irreversible manner perturbing membrane transport [21]. ATP in general and membrane trafficking in particular, as mentioned above, play crucial roles during mitosis and cytokinesis. However, we showed that wiskostatin, at the highest concentration used in this study (10 μM), did not affect cellular ATP levels during mitosis/cytokinesis. Thus, inhibition of cytokinesis induced by wiskostatin is likely independent to its ATP depleting activity.

The existence of additional targets of wiskostatin is supported by the results we obtained using N-WASP mutants. The expression of a dominant negative mutant of N-WASP towards the Arp2/3 complex (N-WASP ΔA) did not block cytokinesis suggesting again that N-WASP inhibition is not sufficient. In contrast, overexpression of N-WASP VCA domain slightly increased nuclei number. The VCA module alters Arp2/3 activity by titrating and delocalising it. VCA overexpression could either block the activation of the Arp2/3 complex by another factor than N-WASP involved in cytokinesis. On the other hand, the increase of F-actin generated by over-activation of Arp2/3 complex triggered by VCA overexpression could affect cytokinesis. This hypothesis is supported by a recent study showing that expression of a constitutively active mutant of WASP towards the Arp2/3 complex increases cell binucleation in a same extend as VCA [19].

## Conclusion

Wiskostatin has been designed, as numerous inhibitors, to specifically inhibit N-WASP activity towards the Arp2/3 complex. Our studies reveal the caveat of using such an *in vitro *approach to identify new compounds. Indeed, *in vivo *test may not parallel the *in vitro *activity and should be included in the compound characterisation. We show that wiskostatin inhibits cytokinesis and it is likely that N-WASP is not its sole target during this process. Our study is the second report of a non-expected effect of wiskostatin, which suggests that this compound will have to be used in future with caution. However, it will be of interest to characterise wiskostatin targets involved in cytokinesis. The identification of these proteins could be achieved using a proteomic approach based upon mass spectrometry using immobilised wiskostatin over mitotic cell extracts.

## Methods

### Reagents and antibodies

All chemicals were purchased from Sigma unless otherwise stated. Monoclonal antibodies against Arp3, β-tubulin (TUB 2.1) and γ-tubulin (GTU-88) were from Sigma. Polyclonal antibody against p34 (ARPC2) was from Upstate Biotechnology. Polyclonal antibodies against N-WASP (H-100), cyclin B1 (GNS1) and ECT2 (C-20) were purchased from Santa-Cruz Biotechnology. Polyclonal antibody against cofilin was from Cytoskeleton. Monoclonal antibody against p-histone H3 (Ser 10) was from Euromedex. Polyclonal antibody against β-tubulin was a generous gift from Jose M. Andreu (Centro de Investigaciones Biológicas, Madrid, Spain). Goat Alexa 350, 488, 555, 680-conjugated anti-mouse and anti-rabbit were from Molecular Probes.

### Expression plasmids

Vector encoding EGFP-tagged bovine N-WASP full-length (aa, 1–505) and ΔA (aa, 1–484) mutant as well as N-WASP CA (aa, 455–505) and VCA (aa, 391–505) regions were from Laura M. Machesky (Beatson Institute, Glasgow, UK).

### Cell culture and synchronisation

HeLa and NIH3T3 cells were cultured in Dulbecco's Modified Eagle's medium (DMEM) supplemented with 10% foetal bovine serum (FBS), 20 mM HEPES and antibiotics. For HeLa cells stably expressing GFP-H2B (gift from E. Julien, IGMM, Montpellier, France) media was supplemented with 0.2 mg/ml G418.

Cells were synchronised in prometaphase by nocodazole block. Briefly cells were grown for 12 hours in the presence of 100 ng/ml nocodazole. Mitotic cells obtained by shake-off were plated onto dishes or glass coverslips coated with poly-L-lysine in fresh growth medium containing vehicle alone or wiskostatin. Cells were then harvested at indicated time points, treated for fluorescence-activated cell sorting (FACS), lysed for immunoblot analysis or fixed for indirect immunofluorescence study. In some experiments, cells were alternatively synchronised in G2/M using 10 μM Cdk1 inhibitor (RO3306, Calbiochem). Briefly, 8 hours after seeding cells were blocked in G1/S with 2.5 mM thymidine for 24 hours. After several washings, cells were released in complete medium containing 24 μM deoxycytine and 10 μM RO3306 for 15 hours. Finally, G2/M blocked cells were released in complete medium containing or not wiskostatin after several washings and treated for immunofluorescence 2 hours after release.

### RNA interference

siRNA were transfected in HeLa cells using Lipofectamine 2000™ reagent (Invitrogen) according to manufacturer instructions. Proteins were targeted with the following sequences: Arp3 with 5'-GCCAAAACCUAUUGAUGUA-3', N-WASP with 5'-GGUGUUGCUUGUCUUGUUA-3' and ECT2 with 5'-CAUUUGAUAUGAAGCGUUA-3'. Cells were harvested 72 hours after transfection for FACS and immunoblot analysis.

### Immnunofluorescence

For immunofluorescence, cells were stained and mounted on glass slides as previously described [40]. In brief, cells were fixed with 4% paraformaldehyde in PEM (0.1 mM PIPES, pH 6.9; 1 mM EGTA; 0.5 mM MgCl_2_) containing 0.2% Triton X-100 for 10 min at 37°C, blocked with 1% BSA and stained with phalloidin or the appropriate antibodies diluted in PBS containing 1% BSA. When mentioned cells were permeabilised with PEM containing 0.5% Triton X-100 and 1 mM PMSF for 30 seconds prior fixation with 4% paraformaldehyde. Cells were examined with a DMR A microscope PL APO 63× oil immersion objective with appropriate filters (Leica) and images were recorded with a cooled CCD Micromax camera (Princeton Instruments) driven by MetaMorph (Molecular Devices).

Time-lapse microscopy was performed using an Axiovert 200 M microscope PlasDIC 32× objective with appropriate filters (Zeiss) and images were recorded with a cooled CCD Micromax camera (Princeton Instruments) driven by MetaMorph (Molecular Devices).

### Determination of cellular ATP levels

1.5 × 10^5 ^mitotic HeLa cells/well in 6 well plates were treated for indicated time with vehicle alone, 10 μM wiskostatin or 10 mM NaN_3 _+ 6 mM 2-deoxyglucose (ATP depletion). Cells were harvested and lysed in boiling distilled water for 10 min. Lysates were clarified by centrifugation at 20,000 × g for 10 min at 4°C. Cellular ATP levels were determined from clarified lysates using ATP determination kit (Proteinkinase, Germany) and following manufacturer instructions. Luminescence was measured using a MicroLumat (Berthold Technologies) luminometer.

### Flow cytometry

Harvested cells were washed in PBS, fixed in 70% ethanol and kept at -20°C until analysis. DNA was stained with propidium iodide and analysed with a FACSCalibur (Becton Dickinson) driven by CellQuest™. The DNA content of 10 000 cells was determined for each condition.

## Abbreviations

WASP: Wiskott-Aldrich syndrome protein; N-WASP: Neural WASP; Arp: actin-related protein; GBD: GTPase binding domain.

## Authors' contributions

GB conceived the study, designed and carried out the experiments and wrote the manuscript. GR assisted with carrying out the experiments. NM assisted in designing the experiments and in writing the manuscript. All authors read and approved the final manuscript.

## Supplementary Material

Additional file 1 **p34 (ARPC2) localisation during the exit of mitosis**. **A**. HeLa cells were enriched in mitosis after thymidine and RO3306 blocks (see Methods for details). Two hours after release from RO3306 block cells were permeabilised and fixed as described in Methods. Cells were treated for indirect Alexa 555 localisation of p34 (a and e) and Alexa 350 localisation of β-tubulin (c and g) with specific antibodies. F-actin was visualised with FITC-coupled phalloidin (b and f). Merged images are presented (d and h). Images are representative of the different stages of mitosis: late telophase (a-d) and cytokinesis (e-h). During late telophase p34 is enriched within the contractile ring (d, arrowhead) During cytokinesis, p34 presented a vesicular-like staining at the basis of microtubule bundles (e, arrowhead) and was found within the cytoplasmic bridge between the two daughter cells (e, arrow). Colocalisation with F-actin was also evident at the cortex facing the cytoplasmic bridge (h, arrowhead). **B**. Arp3 and p34 localisations are undistinguishable HeLa cells prepared as previously described were treated for indirect Alexa 555 localisation of Arp3 (a) and Alexa 488 localisation of p34 (b) with specific antibodies. DNA was stained with DAPI. A merged imaged is presented (d) and highlight the strong colocalisation between the two Arp2/3 complex subunits in particular within the cleavage furrow. Bars, 10 μm. Click here for file

Additional file 2 **Mitosis of vehicle treated HeLa GFP-Histone H2B cells**. For details see figure 2E legend. Frames were taken every 5 min s and are played at six frames per second. Time indicated as hours: minutes. Click here for file

Additional file 3 **Mitosis of wiskostatin (10 μM) treated HeLa GFP-Histone H2B cells**. For details see figure 2F legend. Frames were taken every 5 min and are played at six frames per second. Time indicated as hours: minutes. Click here for file

Additional file 4 **Study of siRNA efficiency**. **A**. Arp3 knockdown affects cell morphology. HeLa cells were transfected with indicated siRNA for 72 hours, permeabilised and fixed as previously indicated and treated for indirect Alexa 555 localisation of Arp3 (a, e and i) using specific antibody. F-actin was stained with FITC-coupled phalloidin (b, f and j). Merged images are presented (c, g and k). Exposure times for Alexa 555 and FITC channels were determined for control conditions and applied to N-WASP and Arp3 null cells allowing direct comparison of protein levels based on signal intensities. Arp3 localised at the tip of membrane protrusion (a and e, arrowhead) and in vesicle-like structures where it colocalised with F-actin (c and g). Arp3 staining is greatly reduced in Arp3 null-cells (compare i with a and e). Bar, 20 μm. **B**. Arp3 recruitment to the contractile ring is not affected in N-WASP null-cells. HeLa in cytokinesis transfected with siRNA as previously described were treated for indirect Alexa 555 localisation of Arp3 (a, e and i) and Alexa 350 localisation of β-tubulin (c, g and k) using specific antibodies. F-actin was stained with FITC-coupled phalloidin (b, f and j). Merged images (d, h and l) are presented. Exposure times in Alexa 555 and FITC channels were fixed as previously described. Arp3 recruitment to the contractile ring is identical between control (a, arrowhead) and N-WASP (b, arrowhead) null-cells but is greatly reduced in Arp3 null-cells (i, arrowhead). Bar, 10 μm. **C**. Time course depletion of N-WASP, Arp3 and ECT2 proteins by RNA interference. HeLa cells were transfected with indicated siRNA and were harvested 24, 48 and 72 hours after transfection. Proteins levels were determined by immunoblot analysis using specific antibodies. The maximum of N-WASP knockdown is achieved after 48 hours transfection, whereas 72 hours are required for Arp3 and only 24 hours for ECT2. Click here for file

Additional file 5 **Mitosis of control siRNA treated cells**. For details see figure 4C legend. Frames were taken every 30 min are played at four frames per second. Time indicated as day: hours: minutes. Click here for file

Additional file 6 **Mitosis of N-WASP siRNA treated cells**. For details see figure 4C legend. Frames were taken every 30 min are played at four frames per second. Time indicated as day: hours: minutes. Click here for file

Additional file 7 **Mitosis of Arp3 siRNA treated cells**. For details see figure 4C legend. Frames were taken every 30 min are played at four frames per second. Time indicated as day: hours: minutes. Click here for file

Additional file 8 **Mitosis of N-WASP + Arp3 siRNA treated cells**. For details see figure 4C legend. Frames were taken every 30 min are played at four frames per second. Time indicated as day: hours: minutes. Click here for file

Additional file 9 **Mitosis of ECT2 siRNA treated cells**. For details see figure 4C legend. Frames were taken every 30 min are played at four frames per second. Time indicated as day: hours: minutes. Click here for file
