# Supplementary figures and images for: Inhibition of cytokinesis by wiskostatin does not rely on N-WASP/Arp2/3 complex pathway
Source: BMC Cell Biol. 2008 Jul 30;9:42. doi: 10.1186/1471-2121-9-42 (PMC2527559; doi:10.1186/1471-2121-9-42)

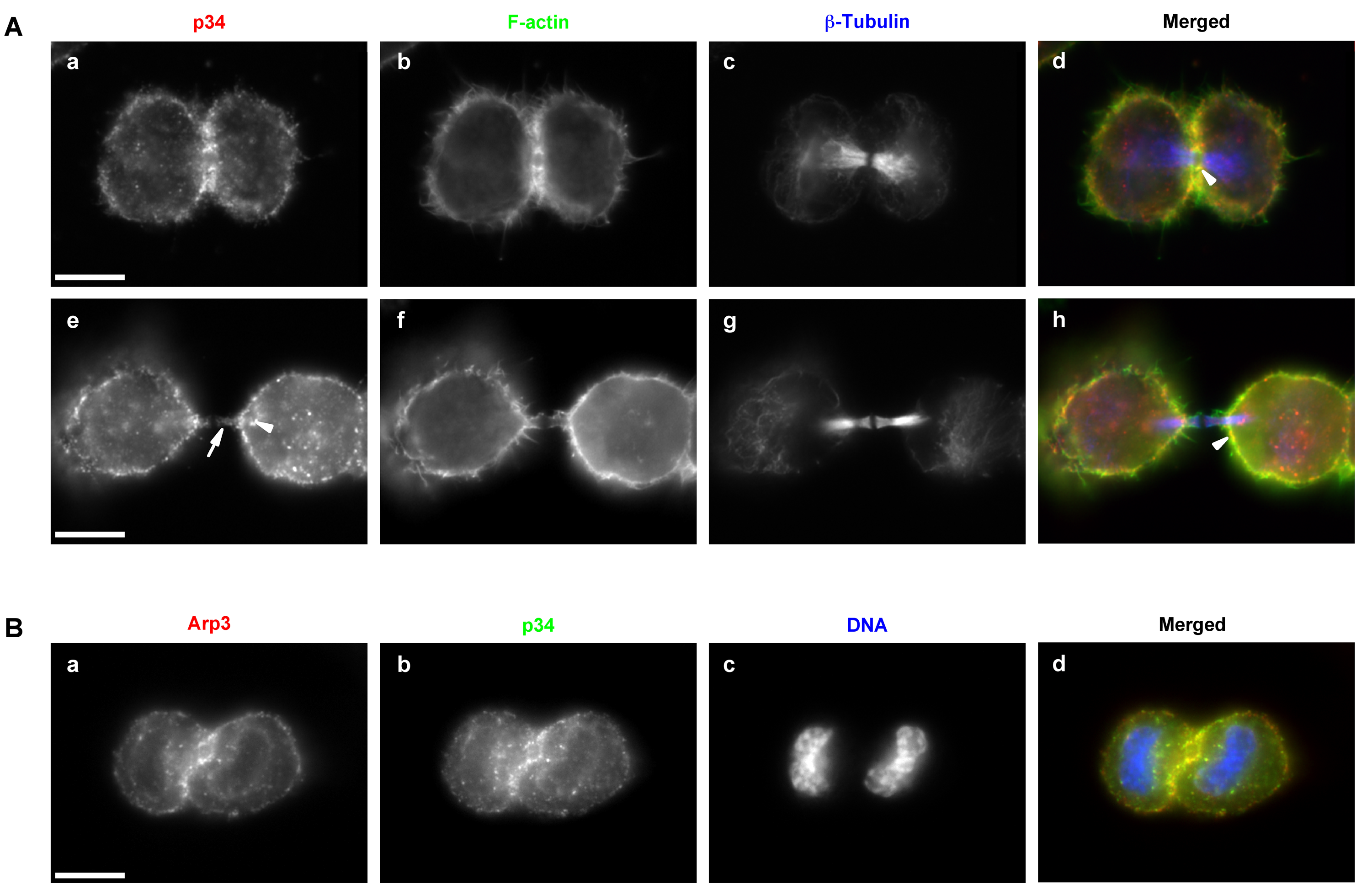

Supplement: Additional file 1 — p34 (ARPC2) localisation during the exit of mitosis. A. HeLa cells were enriched in mitosis after thymidine and RO3306 blocks (see Methods for details). Two hours after release from RO3306 block cells were permeabilised and fixed as described in Methods. Cells were treated for indirect Alexa 555 localisation of p34 (a and e) and Alexa 350 localisation of β-tubulin (c and g) with specific antibodies. F-actin was visualised with FITC-coupled phalloidin (b and f). Merged images are presented (d and h). Images are representative of the different stages of mitosis: late telophase (a-d) and cytokinesis (e-h). During late telophase p34 is enriched within the contractile ring (d, arrowhead) During cytokinesis, p34 presented a vesicular-like staining at the basis of microtubule bundles (e, arrowhead) and was found within the cytoplasmic bridge between the two daughter cells (e, arrow). Colocalisation with F-actin was also evident at the cortex facing the cytoplasmic bridge (h, arrowhead). B. Arp3 and p34 localisations are undistinguishable HeLa cells prepared as previously described were treated for indirect Alexa 555 localisation of Arp3 (a) and Alexa 488 localisation of p34 (b) with specific antibodies. DNA was stained with DAPI. A merged imaged is presented (d) and highlight the strong colocalisation between the two Arp2/3 complex subunits in particular within the cleavage furrow. Bars, 10 μm. [file 1471-2121-9-42-S1.tiff]

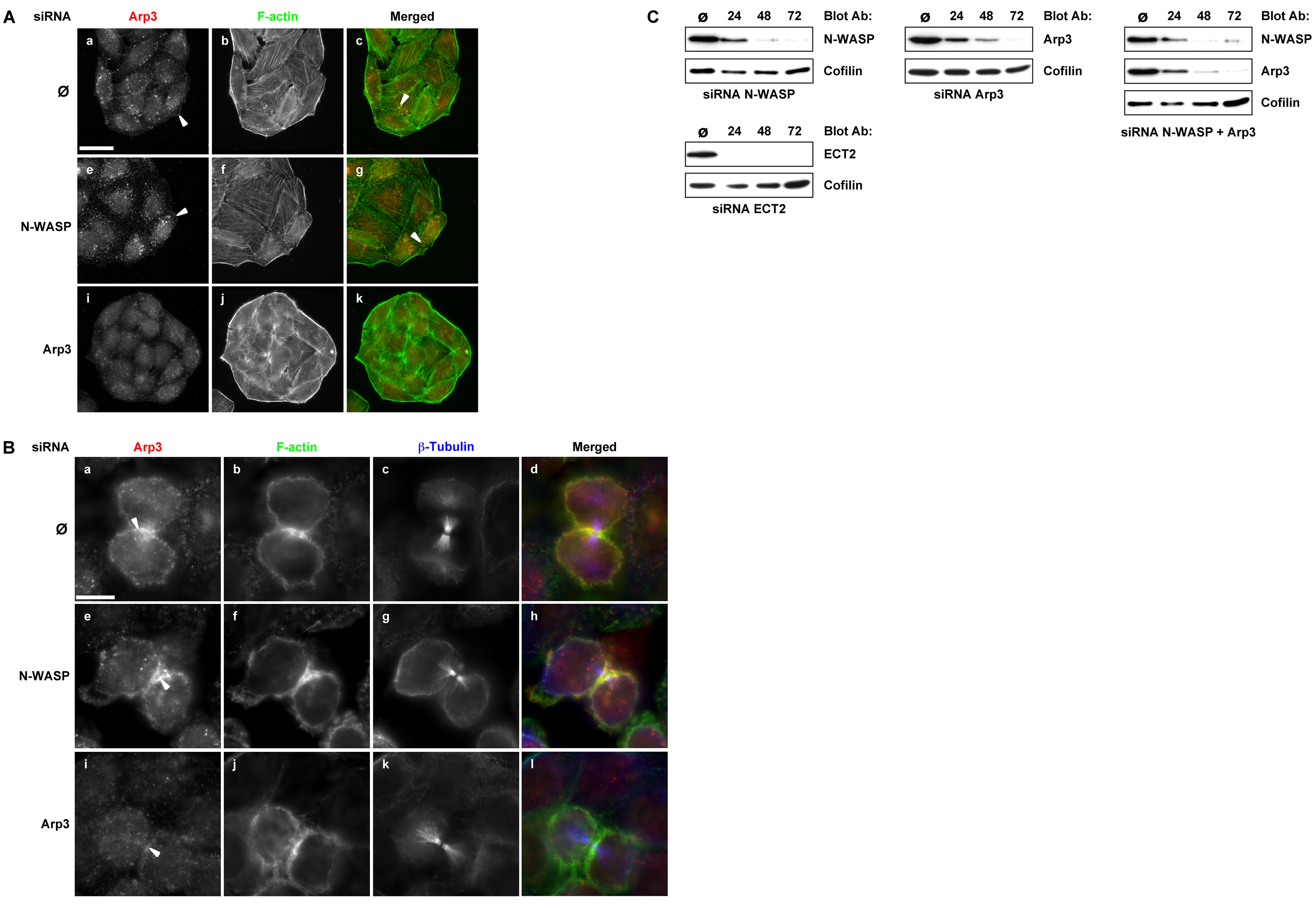

Supplement: Additional file 4 — Study of siRNA efficiency. A. Arp3 knockdown affects cell morphology. HeLa cells were transfected with indicated siRNA for 72 hours, permeabilised and fixed as previously indicated and treated for indirect Alexa 555 localisation of Arp3 (a, e and i) using specific antibody. F-actin was stained with FITC-coupled phalloidin (b, f and j). Merged images are presented (c, g and k). Exposure times for Alexa 555 and FITC channels were determined for control conditions and applied to N-WASP and Arp3 null cells allowing direct comparison of protein levels based on signal intensities. Arp3 localised at the tip of membrane protrusion (a and e, arrowhead) and in vesicle-like structures where it colocalised with F-actin (c and g). Arp3 staining is greatly reduced in Arp3 null-cells (compare i with a and e). Bar, 20 μm. B. Arp3 recruitment to the contractile ring is not affected in N-WASP null-cells. HeLa in cytokinesis transfected with siRNA as previously described were treated for indirect Alexa 555 localisation of Arp3 (a, e and i) and Alexa 350 localisation of β-tubulin (c, g and k) using specific antibodies. F-actin was stained with FITC-coupled phalloidin (b, f and j). Merged images (d, h and l) are presented. Exposure times in Alexa 555 and FITC channels were fixed as previously described. Arp3 recruitment to the contractile ring is identical between control (a, arrowhead) and N-WASP (b, arrowhead) null-cells but is greatly reduced in Arp3 null-cells (i, arrowhead). Bar, 10 μm. C. Time course depletion of N-WASP, Arp3 and ECT2 proteins by RNA interference. HeLa cells were transfected with indicated siRNA and were harvested 24, 48 and 72 hours after transfection. Proteins levels were determined by immunoblot analysis using specific antibodies. The maximum of N-WASP knockdown is achieved after 48 hours transfection, whereas 72 hours are required for Arp3 and only 24 hours for ECT2. [file 1471-2121-9-42-S4.tiff]
